# Review of "Perspectives on Free and Open Source Software" edited by Feller J, Fitzgerald B, Hissam SE and Lakhani KR

**DOI:** 10.1186/1475-925X-5-23

**Published:** 2006-04-05

**Authors:** Saturnino Luz

**Affiliations:** 1 Department of Computer Science, Trinity College Dublin, Dublin 2, Ireland

## 

Feller J, Fitzgerald B, Hissam SE and Lakhari KR (Editors)

Perspectives on Free and Open Source Software

Cambridge, MA: MIT Press, 2005

538 pages, ISBN 0-262-06246-1

*Perspectives on Free and Open Source Software *is a collection of articles by researchers, advocates and critics of the Free and Open Source Software (FOSS) movement and its way of building and distributing software. The book represents a serious attempt to assess the current state of FOSS and discuss its future.

The volume is structured along 5 parts, some containing cohesive sets of articles, some loosely related ones. The three chapters that make up part 1 report on attempts to explain the FOSS phenomenon from the perspective of the psychology and sociology of its main actors. In particular, these articles try to explain what leads FOSS developers not to sell the software they create, but rather regard that software as *free*. The question appears to be framed initially in terms of explaining "free as in *free beer*" rather than "free as in *freedom*" [1], presumably in order to keep the attention of the business reader. However, detailed investigation soon reveals the complexities of the issue and the inadequacy of that frame of reference. Part 2 discusses issues relating to FOSS evaluation, where the term "evaluation" is used in a broader sense than in software engineering. The term here designates an attempt to ascertain not only the value of open source as a development methodology but its worth and long-term prospects as a movement. Three of the chapters address technical questions, including interesting discussions of the effects of public access to source code on security. The remaining three chapters contain analyses and (at times extravagant) responses to (similarly extravagant) claims about the inherent superiority of FOSS found in the literature, particularly those by Raymond [2]. Part 3 counters the impression left by part 2 that software engineering these days is mostly about politics, economics and anthropology by presenting actual software engineering tools and processes used in FOSS development. Part 4 returns to economics and introduces a characterisation of FOSS as a phenomenon typical of markets driven by user innovation, discusses business models which incorporate elements of FOSS, and voices opinions of software vendors on such models. Finally, part 5 bundles together articles on legal aspects of FOSS and its social implications. This is the least cohesive but possibly the most interesting part of the book in that it discusses, though to a limited extent, the underlying philosophy of FOSS and the social and cultural conflicts that oppose it to the *status quo*. Part 5 also includes an enlightening legal analysis of FOSS licenses, focusing on the most famous of them, the General Public License (GPL), and chapters on broader cultural, economic and policy issues, such as the increasingly important topic of the role FOSS plays and might play in the governmental and public sectors.

Although the book encompasses several perspectives, it appears to target primarily managers, particularly those who feel they ought to learn more about FOSS in order to be able to draw advantage from it. The predominance of articles on business models and open-source as a potentially superior development methodology over articles on the political and philosophical background of free software seems designed to give the book an air of business friendly pragmatism. While this might serve to allay instinctive fears business readers might feel in relation to FOSS, it also has the effect of making the text seem at times rather dull (loaded with economic jargon) to the more technically-minded reader such as myself and, I suspect, "not dull enough" (detailed, backed by extensive data and analysis) to book's target audience. Moreover, by attempting to avoid the pitfalls of ideology, many of the articles in the book end up making tacit ideological assumptions. One such assumption is explicitly stated in the preface: "The business of selling software products will live on, along with free and open source programs. This is most likely how it will be, and it is how it should be." Tacit adherence to premises such as this might have contributed to narrowing the scope of the book beyond what is needed to analyse a movement the editors describe in the introduction as "a revolution".

Attaining objectivity in an incipient field fraught with controversy, passionate stances and conflicting interests is an ambitious goal. Despite the above mentioned shortcomings, the book makes a welcome contribution toward achieving that goal.
